# Serotype distribution and antimicrobial susceptibility of *Streptococcus suis* isolates from porcine diagnostic samples in Hungary, 2020–2023

**DOI:** 10.1186/s40813-024-00419-0

**Published:** 2025-01-08

**Authors:** Ervin Albert, István Emil Kis, Krisztián Kiss, Katalin K-Jánosi, Tamás Révész, Imre Biksi

**Affiliations:** 1 Department of Pathology, University of Veterinary Medicine Budapest, Üllő, Hungary; 2 Institute of Metagenomics, University of Debrecen, Debrecen, Hungary; 3 SCG Diagnosztika Kft., Délegyháza, Hungary; 4 Ceva-Phylaxia Zrt., Budapest, Hungary

**Keywords:** *Streptococcus suis*, Serotype, *Cps-*type, Antimicrobial susceptibility, AMR, Antimicrobial resistance profile, Resistotype

## Abstract

**Background:**

*Streptococcus suis* (*S. suis*) is a major swine pathogen and a significant zoonotic agent, causing substantial economic losses in the swine sector and having considerable public health importance. The control and management of *S. suis*-related conditions has become increasingly challenging due to the multitude of involved serotypes with varying antimicrobial resistance patterns. Here, we report the serological distribution and antimicrobial susceptibility of *S. suis* isolates isolated form clinical samples of Hungarian large-scale swine farms.

**Results:**

Between 2020 and 2023, altogether 296 *S. suis* isolates were obtained from diseased pigs of 64 Hungarian pig operations. Serotyping of the isolates was carried out by using molecular methods (*cps*-typing). The isolated strains belonged to 24 single *cps*-types. The most frequently detected *cps-*types during the four years of this passive survey were 9 (19.6%), 2 (19.3%), 1/2 (18.9%) and 7 (14.5%). The brain, spleen, endocardial valve thrombus and lung proved to be the most frequent site of *S. suis* strain isolation, and animals 29–75 days of age were affected in the highest proportion.

Antimicrobial susceptibility testing of the isolates was performed by determining the minimal inhibitory concentration for 15 antimicrobial agents of veterinary and human importance using a commercial microdilution assay. More than 90% of the tested isolates proved to be susceptible to the examined beta-lactams, cephalosporins and florfenicol, as well as to rifampicin, trimethoprim/sulfamethoxazole and vancomycin. Phenotypic resistance profiles (resistotypes) of clindamycin-tetracyclin (3.8%), clindamycin-erythromycin-tetracyclin (8.4%) and clindamycin-erythromycin-tetracyclin-trimethoprim / sulfamethoxazole (3.8%) were most frequently detected. Vancomycin resistance was observed in the case of 1 *S. suis* strain.

**Conclusions:**

The dominance of *S. suis cps*-types 9, 2, 1/2 and 7 in Hungary over the four years of this study aligns with previous reports from several countries worldwide. The presence of highly susceptible *S. suis* isolates suggests a prudent antibiotic usage and treatment practice in the surveyed Hungarian swine operations. In contrary, the presence of several resistotypes could indicate the problem of antibiotic resistance in the future.

**Supplementary Information:**

The online version contains supplementary material available at 10.1186/s40813-024-00419-0.

## Background

*S. suis* is one of the major swine pathogens and an important emerging zoonotic agent [1, 2]. It was first reported in the 1950s causing meningoencephalitis and arthritis in piglets [3, 4] and was formally named as new species *S. suis* in 1987 [5]. Since then the bacterium has been reported globally in both extensive and intensive swine producing farms, causing serious economic losses in the sector [1].

*S. suis* is a natural inhabitant primarily of the upper respiratory (nasal cavities and tonsils), the lower genital and gastrointestinal tract of pigs [6, 7], therefore it is quite easy to detect from almost all pigs of any age [8]. Nearly all sows carry *S. suis* strains on their vaginal mucosa [9]. In addition to vertical transmission during farrowing [9], the bacterium can also be transmitted through direct, indirect and airborne routes [10]. Temperature fluctuation, high relative humidity, overcrowding and large age gaps between groups proved to be predisposing factors for increasing the carrier state of *S. suis* in pigs [11, 12].

*S. suis* can cause meningitis, polyserositis, arthritis, valvular endocarditis and pneumonia mainly during the postweaning period, as well as septicaemia leading to sudden death of 5–10 week-old pigs [1, 6, 13].

*S. suis* strains can be serologically classified on the basis of their capsular polysaccharide (CPS) antigens [14]. In total 35 serotypes (1–34 and 1/2) have been reported between 1966 and 1995. Serotypes 20, 22 and 26 were recently reappraised as novel species *S. parasuis* as well as serotypes 32 and 34 are considered now as *S. orisratti*, on the basis of molecular approaches [15–18], and a novel *Chz* serotype was also proposed [19]. CPS switching of *S. suis* from serotype 2 to 3, 4, 7, 8, 9 or 14 was experimentally demonstrated by full *cps* locus exchange, that indicates the possible serotype switching amongst different *S. suis* isolates [20].

Most *S. suis* infections in pigs are related to serotypes 2 and 9, but the predominant serotypes causing invasive disease in pigs can vary [21, 22]. Several studies confirmed serotype 2 either alone [23–26] or together with serotype 1/2 [27] to be the most prevalent among *S. suis* isolates, and serotypes 3, 4, 5, 7 and 8 are also became more frequently detected [28]. The incidence of serotype 3 detection had decreased, while that of serotype 9 and 14 increased in recent years [22, 27–29]. Geographical distribution of serotypes showed similar pattern in Denmark, France, Italy, Spain and Japan with serotypes 9 and 2 dominance [21–25]. Recent data revealed serotype 7 as the most frequent amongst Czech isolates [30].

*S. suis* is an emerging zoonotic pathogen and *serotype* 2 proved to be the most common cause of human cases [6] while rare serotypes, like 16 was also reported from a fatal human case [31].

Despite the widespread use of co-agglutination test for CPS serotyping in laboratory diagnostics [32], it is not discriminative enough to precisely differentiate *S. suis* strains [33], because of hydrophobic properties of certain strains as well as the presence of cross- and autoagglutination [28]. Because of the difficulties of agglutination tests, molecular methods such as capsular-typing (*cps-*typing) by polymerase chain reaction (PCR) are widely used as an alternative approach for determining the serotype in the diagnostics of *S. suis* [34].

Control of *S. suis* infection is a source of concern for all participants of the swine industry [1], not only because of its negative economic consequences but also because of the zoonotic potential of the pathogen [2, 35]. In several countries, the control of *S. suis* infection is based on the use of antimicrobials, although the routine application of them contributes to the development of antimicrobial resistance (AMR) [21] and as a resistance reservoir, *S. suis* could play a role in the spread of AMR genes [36]. High rates of phenotypic resistance of *S. suis* isolates to lincosamides, macrolides, tetracyclines and sulphonamides were documented worldwide [37–39]. The high prevalence of the combined phenotypic manifestation of the above mentioned resistance profiles (resistotypes) of *S. suis* strains draws attention to the importance of continuous AMR surveillance of the pathogen [39, 40].

The resistance of the bacterium to aminoglycosides, beta-lactams, chloramphenicol, fluoroquinolones and trimethoprim-sulfamethoxazole was also reported [41–46], and a global tendency in increase of AMR in *S. suis* to aminoglycosides, cephalosporins, fluoroquinolones, macrolides, tetracycline and vancomycin could also be observed [39, 47–50].

Here we report the serological and AMR distribution of clinical *S. suis* isolates from Hungarian swine farms of different sizes. Our aim was to identify the most important serotypes causing clinical disease in Hungarian pig herds, to help the efficient selection of targets for autovaccines. We also would like to determine the most common phenotypic antimicrobial resistance profiles and assist in antimicrobial selection through reporting cumulative AMR data. This would be valuable information if an *S. suis* antibiotic susceptibility testing is not yet available for a particular herd.

## Methods

### *S. suis* isolates

**Fig. 1:**
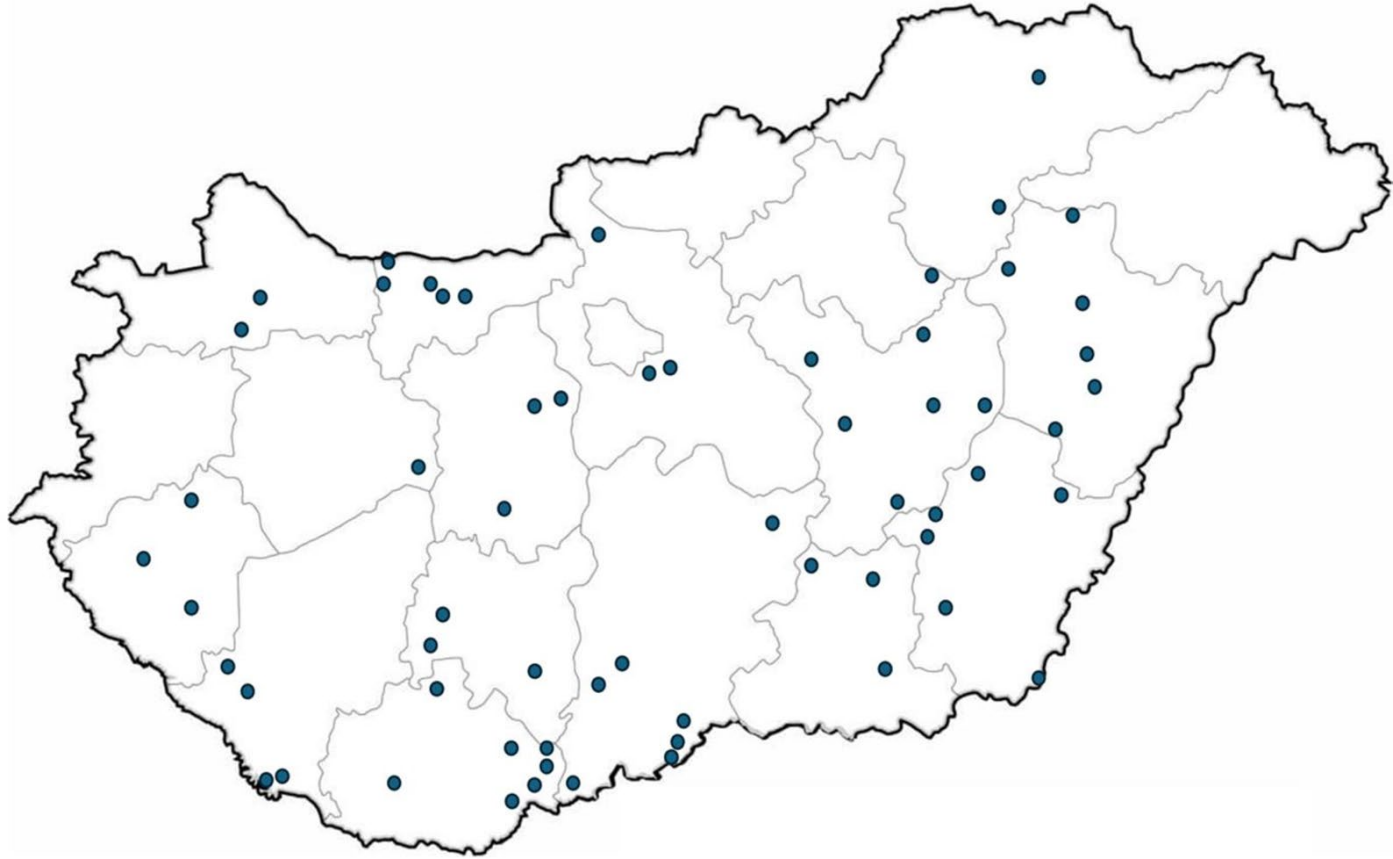
Geographical distribution of Hungarian swine farms involved in the study. Overall, 62 points are indicated on the map, because two farms are at the same settlement, and the origin of 1 sample in 2021 is unknown

Diagnostic samples were collected from 64 large-scale swine farms (counted at least 500 sows and their progeny, either reared farrow-to-finish or as multisite) in Hungary (farm data are anonymised, see Fig. 1) between 1^st^ January 2020 and 31^st^ December 2023. Organ samples or respective swab samples and cadavers were sent to the Livestock Diagnostic Centre (Department of Pathology, University of Veterinary Medicine Budapest, Üllő, Hungary) for laboratory diagnostics.

Samples were distinguished according to their organ origin and were assigned into four age groups (0–28 days—pre-weaning group; 29–75 days—growers; 76–180 days—fatteners; 180 + —breeding sows).

Routine aerobic bacterial examination of the diagnostic samples was carried out on Columbia 5% sheep blood agar (Biolab, Budapest, Hungary) at 37 ± 1 °C for 24–48 h under normal atmospheric conditions. Colonies from primary cultures of presumptive streptococci were sub-cultured and identified on genus level using Gram-staining and basic biochemical tests. The identification on species and subspecies level was carried out using API 20 Strep biochemical test kit (bioMérieux, Belgium). Isolates were stored at -80°C until further investigation.

Altogether 296 *S. suis* isolates were obtained. Yearly distribution of the isolates is summarized in Table 1.

**Table 1:** Yearly distribution of *S. suis* isolates

Year of isolation	No. of isolates	No. of farms of isolation
2020	55	16
2021	74	25
2022	68	24
2023	99	35

### Capsular typing of *S. suis* isolates

Bacterial nucleic acid was isolated from pure cultures of all isolates on an ongoing basis by using a direct lysis method, according to the manufacturer’s instructions (PrepMan Ultra, Thermo Fisher Scientific, Warrington, United Kingdom). For the detection of *cps*-types, a multiplex PCR system was implemented [34]. The PCR tests contained all primers for each recognized *S. suis cps*-type and a primer set for the *S. suis*-specific glutamate dehydrogenase (*gdh*) sequence, the latter intended for species confirmation [34]. *Cps*-types 1/2 and 2 as well as 1 and 14 were discriminated by using a mismatch amplification mutation assay PCR as previously described [51]. Since the *gdh* assay may detect closely related *Streptococcus* spp. as well [34], isolates without detectable *cps*-type were confirmed to be *S. suis* applying a PCR proved to be more species-specific than the one incorporated into the multiplex *cps*-typing assay [52]. Isolates with uncertain or negative PCR results were sub-cultured a second time and re-tested (data not shown).

### Antimicrobial resistance testing of *S. suis* isolates

Antimicrobial susceptibility testing of *S. suis* strains (n = 287) was performed by determining the minimal inhibitory concentration (MIC) for 15 antimicrobial agents with a commercial microdilution assay (MICRONAUT-S Lifestock/Equines GP, E1-299; Merlin Diagnostika GmbH, Berlin, Germany). Assays were carried out according to the manufacturer’s instructions. Breakpoints for ampicillin, cefquinome, ceftiofur, cefazolin, clindamycin, cefoxitin, enrofloxacin, erythromycin, florfenicol, gentamicin, penicillin G, rifampicin, trimethoprim/sulfamethoxazole, tetracycline and vancomycin to clinical categories were assigned automatically by the manufacturer’s software according to Clinical and Laboratory Standards Institute (CLSI) standards that were valid at the time the plates were defined. For antibiotics not listed in the above mentioned CLSI standards, the susceptible-intermediate-resistant (SIR) assessment was carried out in accordance with the recommendations of the Working Group on Antibiotic Resistance, German Veterinary Medical Society (DVG).

The full list of the antimicrobial compounds and the number of successfully tested *S. suis* isolates can be found in Table 2. Of the isolated 296 *S. suis* strains, a total of 287 could be successfully examined, due to laboratory technical failures or software analytical issues. In the case of clindamycin, cefoxitin, enrofloxacin, erythromycin and tetracycline further technical issues occurred and resulted the reduction of the number of the examined strains. Repeated testing of such isolates was not readily possible because the manufacturer had recently discontinued the production of the commercial microdilution assay used in our study.

Phenotypic antimicrobial resistance profiles (resistotypes) were constructed based on resistance patterns. Resistotypes containing more than 6 antimicrobial agents as well as the presence of ampicillin resistance without penicillin G resistance are considered as technical errors and were excluded from the investigations.

**Table 2:** Summarized data of AMR tests

Antimicrobial agent	Range of observation (mg/L)	Number of *S. suis* isolates				
		2020–2023	2020	2021	2022	2023
Ampicillin	*0.125–8*	287	53	74	68	97
Cefquinome	*2–4*	287	53	74	68	97
Ceftiofur	*0.125–4*	287	53	74	68	97
Cefazolin	*1–8*	287	53	74	68	97
Clindamycin	*0.125–2*	277	53	74	58	96
Cefoxitin	*2–4*	286	53	74	67	97
Enrofloxacin	*0.0625–2*	286	53	74	67	97
Erythromycin	*0.125–4*	287	53	74	68	97
Florfenicol	*1–8*	284	53	74	67	95
Gentamicin	*1–8*	287	53	74	68	97
Penicillin G	*0.125–8*	287	53	74	68	97
Rifampicin	*0.0625–4*	287	53	74	68	97
Trimethoprim/sulfamethoxazole	*0.25/4.75* *4/76*	287	53	74	68	97
Tetracycline	*0.25–8*	286	53	74	68	96
Vancomycin	*0.5–16*	287	53	74	68	97

## Results

### Capsular type, organ and production group distribution of *S. suis* isolates

Between 2020 and 2023, 24 single capsular-types (*cps*-types) were found during the examination of the 296 *S. suis* isolates (Table 3). Capsular types of 1/2 or 2 and 14 or 8 could not be distinguished in 1.69% and 0.34% of the samples, while in 1.35% of the cases *cps-*types could not be identified by the applied molecular methods [34], although the isolates were confirmed to be *S. suis*. *Cps-*types 9 (19.6%), 2 (19.3%), 1/2 (18.9%) and 7 (14.5%) were detected most frequently during the 4 years of the passive survey. All other 20 detected single *cps*-types were identified in less than 3.5% of the isolates, moreover 11 of them were found in less than 1% of the cases (Table 3).

**Table 3:** Organ and cps-type distribution of *S. suis* isolates between 2020 and 2023

*cps-*type	2020–2023 (%)	Brain	Spleen	Endocardial thrombus	Lung	Joint	Pericardium	N/A	Vaginal swab	Heart blood swab	Tonsil
*9*	58 (19.6)	20	18	9	7	1	2	–	1	–	–
*2*	57 (19.3)	15	13	17	8	1	2	1	–	–	–
*1/2*	56 (18.9)	17	23	9	5	1	1	–	–	–	–
*7*	43 (14.5)	14	9	7	5	5	3	–	–	–	–
*3*	10 (3.4)	5	4	–	1	–	–	–	–	–	–
*1*	9 (3.0)	2	3	2	–	2	–	–	–	–	–
*4*	9 (3.0)	2	3	1	1	–	–	2	–	–	–
*8*	8 (2.7)	2	2	1	–	–	3	–	–	–	–
*16*	7 (2.4)	–	1	3	–	3	–	–	–	–	–
*14*	5 (1.7)	–	1	4	–	–	–	–	–	–	–
*1/2 or 2*	5 (1.7)	–	–	3	–	–	1	–	1	–	–
*10*	4 (1.4)	–	–	1	1	1	–	1	–	–	–
*-*	4 (1.4)	1	2	–	1	–	–	–	–	–	–
*21*	3 (1.0)	–	–	–	1	2	–	–	–	–	–
*31*	3 (1.0)	–	–	–	1	2	–	–	–	–	–
*5*	2 (0.7)	–	–	–	2	–	–	–	–	–	–
*6*	2 (0.7)	–	–	–	–	2	–	–	–	–	–
*24*	2 (0.7)	1	1	–	–	–	–	–	–	–	–
*11*	1 (0.3)	–	–	–	–	1	–	–	–	–	–
*12*	1 (0.3)	–	–	1	–	–	–	–	–	–	–
*15*	1 (0.3)	–	–	–	–	–	–	–	–	–	1
*18*	1 (0.3)	–	–	–	1	–	–	–	–	–	–
*19*	1 (0.3)	–	–	–	1	–	–	–	–	–	–
*25*	1 (0.3)	1	–	–	–	–	–	–	–	–	–
*28*	1 (0.3)	1	–	–	–	–	–	–	–	–	–
*29*	1 (0.3)	–	–	–	–	–	–	–	–	1	–
*14 or 8*	1 (0.3)	1		–	–	–	–	–	–	–	–
SUM	296	**82**	**80**	**58**	**35**	**21**	**12**	**4**	**2**	**1**	**1**

N/A – not applicable. Numbers in brackets are given in percentage

In total 11, 14, 13 and 17 single *cps-*types were detected in 2020, 2021, 2022 and 2023, respectively. Between 2020 and 2023 *cps*-types 1/2, 9 and 2 were identified in the highest proportion of the isolates*.*

Regarding the organ sites of isolation, the highest proportion of *S. suis* strains were isolated from brain (27.7%), spleen (27.0%), endocardial valve thrombus (19.6%) and lung (11.8%) samples. In the case of lung samples, it should be considered that the isolation of *S. suis* might be a consequence of contamination from the upper respiratory tract. Only 7.1% of the isolates were cultured from synovial exudate samples. *Cps-*types 9 and 7 were most frequently isolated from brain samples (34.5% and 32.6%), *cps-*type 2 from endocardial valve thrombus (29.8%), and *cps-*type 1/2 from spleen (41.1%) samples.

Regarding the age groups 9.5%, 68.2%, 13.2% and 1.4% of the samples originated from the 0–28 days-old (pre-weaning), the 29–75 days-old (growers), the 76–180 days-old (fattener) and 180 days + (breeding sows) group, respectively. In case of 8% of the samples no age groups were assigned. The most frequent organ sites of origin of *S. suis* isolates in the two younger age groups (0–28 and 29–75 days), were brain and spleen (35.7% and 28.6%; 31.2% and 29.7% respectively), while in the finisher facility (76–180 days) 46.2% of the strains originated from endocardial valve thrombuses.

Assessing the frequency of *cps-*types in a certain age group, it was observed that *cps-*types 1/2 and 7 (both 17.9%) were the most frequent in the pre-weaning period (0–28 days). In the youngest age group 14.3% of the isolated *S. suis* strains were *cps-*type 16, while only 3.6% of the isolates were *cps-*type 2 and *cps-*type 9 was not detected at all. Altogether 20 single *cps*-types were detected from organ samples of pigs of 29–75 days of age. During the growing (29–75 days) and the fattening period (76–180 days) *cps-*types 2, 1/2 and 9 (19.3%, 19.8% and 20.8%; 33.3%, 25,6% and 15.4% respectively) were predominant, while *cps-*type 7 was also frequently detected (16.3%) in the post weaning period.

### Results of antimicrobial resistance testing of *S. suis* isolates

More than 90% of the tested isolates proved to be susceptible to the examined penicillin-derivates (ampicillin, penicillin G), cephalosporins (cefquinome, ceftiofur, cefazolin, cefoxitin), florfenicol as well as to rifampicin, trimethoprim/sulfamethoxazole, and vancomycin (Table 4). The percentage of susceptible strains to erythromycin and to clindamycin was 76.6% and 71.8%, respectively. In case of gentamicin and enrofloxacin, 68.3% and 66.1% of the isolates proved to be susceptible. Only 55.9% of the isolates showed susceptibility to tetracycline and one appeared to be resistant to vancomycin (MIC = 2 μg/mL).

**Table 4:**
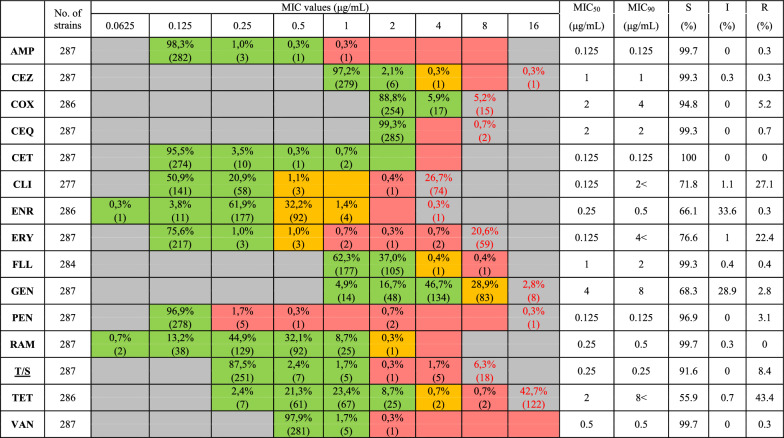
MIC distribution and AMR results of *S. suis* isolates (2020–2023)

AMP—ampicillin; CEQ—cefquinome; CET—ceftiofur, CEZ—cefazolin, CLI—clindamycin, COX—cefoxitin, ENR—enrofloxacin, ERY—erythromycin, FLL—florfenicol, GEN—gentamicin (normal), PEN—penicillin G, RAM—rifampicin, T/S—trimethoprim/sulfamethoxazole, TET—tetracycline, VAN—vancomycin; S—susceptible; I—intermediate; R—resistant; Red cells—resistant, yellow cells—intermediate, green cells – susceptible

Red, yellow and green cells indicate the dilution range tested

Values in the grey cells (red font colour) indicate MIC values over the highest concentration of the tested range

T/S was tested in a fixed 1:5 ratio, MIC values represent the trimethoprim concentration in the table

A single-peaked distribution of MIC values was observed in case of all examined antimicrobial agents except for clindamycin, erythromycin and tetracycline where the MIC values showed bimodal distribution.

Assessing the resistotypes of the examined *S. suis* strains, the clindamycin-erythromycin-tetracyclin combinate profile was found in 8.4%, and proved to be the most frequent resistance pattern.

The combination of clindamycin and erythromycin resistance could be observed in 19.1% of the isolates, while clindamycin and tetracycline resistance together were found in 22.3% of the strains. Strains having CLI-ERY-TET resistotype were isolated from the age group of 29–75 days in 58.3% of the cases.

The resistotypes that could be observed in at least 1.0% of the strains are shown in Table 5.

**Table 5:** Resistotypes of the examined *S. suis* isolates

Resistotype	%
TET	17.4
CLI-ERY-TET	8.4
CLI-TET	3.8
CLI-ERY-TET-T/S	3.8
CLI-ERY	2.1
ERY-TET	2.1
CLI	1.7
ERY	1.4
GEN	1.4
COX-TET	1.4
CLI-ERY-TET-T/S-COX	1.0

Resistotypes that could be observed in more than 1% of the examined strains are shown

## Discussion

*S. suis* is an increasingly significant opportunistic pathogen of pigs and has been reported to cause severe economic losses in the swine industry [23, 24, 27]. Determining the serotype or the *cps-*type of the isolated strains is crucial in understanding the epidemiology of the bacterium. Monitoring the antimicrobial susceptibility profiles of *S. suis* is also of great importance, given that this zoonotic pathogen is considered as a reservoir for antibiotic resistance and represents a high risk of transmission of resistance to other pathogens [49].

All *S. suis* strains in this study were isolated from tissues of diseased pigs exhibiting various lesions, with the brain and spleen being the most common organs of isolation. Since only organs with alterations were sampled in our passive survey, the detected bacteria most likely had contributed to the observed lesions. These data, however, are not aimed to indicate that the sole underlaying pathogen was *S. suis* of the experienced clinical and pathological conditions. This is especially true for the lungs, where the relevance of *S. suis* as a pathogen is still controversial. The bacterium is commonly found in the upper respiratory tract, and the mere presence of potentially virulent isolates does not necessarily lead to the manifestation of clinical symptoms [53].

The carrier rate of the *S. suis* is usually very high [54], and individual pigs are often colonized by multiple serotypes [55]. *Cps-*types 9, 2, 1/2 and 7 were detected most frequently, accounting for 72.3% of the isolates. The consistent presence and the high detection rate of *cps*-type 2 was observed, similar to previous findings in Japan [23], Denmark [45], Belgium, France, Germany, Italy, Spain, the Netherlands, the UK [25], Canada [27] and in the Czech Republic [30]. *Cps-*type 9 proved to be the most prevalent serotype between 2020 and 2023 in Hungary. It has also emerged as an invasive and predominant *cps-*type in Switzerland [56, 57], Spain, Germany and in the Netherlands [21], despite it was formerly considered as a largely subclinical colonizer of the mucous membranes of swine [58]. A *cps-*type shift could be observed on a yearly basis: *cps-*type 1/2 was the predominant type in 2020 and in 2021, while *cps*-type 9 could be detected in most of the isolated strains in 2022 and 2023. The increase in the predominance of *cps*-type 9 has also been described previously [27–29]. The spread of *S. suis cps-*type 9 could be facilitated by the selective advantage gaining due to the lack of immunity, induced by the widespread presence of *cps-*type 2 [59] and by the higher level of bacterial protection provided by the CPS of *cps-*type 9 [60].

Even though swollen joints and arthritis are important clinical signs and lesions of *S. suis* disease [6, 61], only 7.1% of the isolates were isolated from joints in our survey. This aligns with observations reported from the Czech Republic, where only 5.0% of the isolates originated from joints [30].

The vast majority (74.0%) of the *S. suis* strains were isolated from samples of animals between 29 and 75 days of age, which correlates with the literature data, indicating that diseased animals are usually between 5 and 10 weeks of age [33, 62]. In this age group, *cps-*type 9 proved to be the predominant serotype. Interestingly, *cps-*type 9 was not detected at all in the youngest age group (0–28 days), but a rare and potentially zoonotic *cps-*type 16 was detected in 14.3% of the cases [31, 63].

Along with previous studies [23, 25], the diseased brain (meninges and CNS as well) proved to be the most prevalent organ site of *S. suis* isolation in our survey. Isolation of *S. suis* form lungs was much less frequent during the four years of our survey, than it was previously reported [23–25, 30].

Despite several of the antimicrobial agents included in this commercial panel are neither used against streptococci nor in treatment of swine, such extensive AMR pattern of clinical *S. suis* isolates may have relevance e. g. in human medicine due to the zoonotic character of the pathogen. However, the high percentage of susceptible *S. suis* strains in our AMR examinations indicates a prudent practice of antibiotic treatment in the examined subset of Hungarian swine farms. The choice of antibiotics to treat *S. suis* disease should be based on the knowledge of local AMR patterns [61]. According to our results, beta-lactams, like penicillin and cephalosporins and other frequently used compounds, such as florfenicol, as well as trimethoprim/sulfamethoxazole could be useful antimicrobials for the treatment of *S. suis-*related conditions.

The presence of high percentage of *S. suis* isolates resistant to tetracyclines, macrolides and lincosamides was observed in our study as it was described previously [39, 40, 43–45]. A much lower degree of gentamicin resistance was formerly observed [44] than it was found in our study. It is important to highlight that differences in antimicrobial usage among countries may contribute to apparent differences in antimicrobial resistance patterns.

In treating multidrug-resistant human Gram-positive bacterial infections, vancomycin is one of the critical last-line antimicrobial agents to use. In the last few years, a slight reduction could be observed in the susceptibility to vancomycin [47, 64–66]. According to our results only one *S. suis* strain appeared to be phenotypically resistant to vancomycin, but the result should be interpreted with caution, as the MIC value is located at the borderline between the susceptible and resistant zone. Further confirmatory tests for potential vancomycin resistance could not be performed, as the isolate did not survive storage.

Although resistance genes were not examined in our survey, the phenotypic antimicrobial resistance data suggests the necessity of further genetic investigations. Data obtained in our passive survey underscore the importance of profiling *S. suis* isolates from pigs to monitor antimicrobial resistance trends and facilitate the early identification of emerging clones.

## Conclusions

Our study confirmed the predominant presence of *S. suis cps-*types 2, 9, 1/2 and 7 on large-scale swine farms in Hungary. We have also detected increased occurrence of *cps-*type 9 in Hungary. A rare and potentially zoonotic *cps-*type 16 detected in pre-weaning pigs might raise to public health concern in case of farrowing house workers.

The high percentage of susceptible *S. suis* isolates in AMR tests may indicate prudent antibiotic practices on the examined Hungarian swine farms. However, the presence of isolates with CLI-ERY-TET resistotype, as well as of the presence of a probably vancomycin resistant isolate underline the possibility of challenges in antimicrobial treatment of *S. suis* infections. Further investigations should be carried out to determine the genetic background behind the observed resistance profiles of recent Hungarian *S. suis* isolates.

## Supplementary Information

Additional file 1.

## Data Availability

The data supporting the conclusions of this article are included within the article and in the supplementary material. The data used and/or analyzed during the current study are available from the corresponding author on reasonable request.

## Abbreviations

AMR: Antimicrobial resistance
CLSI: Clinical Laboratory Standards Institute
CPS: Capsular polysaccharide
*cps-*type: Capsular-type
DVG: German Veterinary Medical Society
*gdh*: Glutamate dehydrogenase
ICE: Integrative and conjugative elements
MIC: Minimal inhibitory concentration
PCR: Polymerase chain reaction
*S. suis*: *Streptococcus suis*
SIR: Susceptible-intermediate-resistant

## References

[1] Segura M, Fittipaldi N, Calzas C, Gottschalk M. Critical streptococcus suis virulence factors: are they all really critical? Trends Microbiol. 2017;25:585–99. 28274524 10.1016/j.tim.2017.02.005

[2] Feng Y, Zhang H, Wu Z, Wang S, Cao M, Hu D, et al. Streptococcus suis infection. Virulence. 2014;5:477–97. 24667807 10.4161/viru.28595 PMC4063810

[3] Jansen E, Van Dorssen C. Meningoencephalitis bij varkens door streptococcen. Tijdschr Diergeneeskd. 1951;76:815–32.

[4] Field HI, Buntain D, Done JT. Studies on piglet mortality. I. Strep-tococcal meningitis and arthritis. Vet Rec. 1954;66:453–5. 10.5555/19552200564.

[5] Kilpper-Balz R, Schleifer KH. Streptococcus suis sp. Nov., nom. rev. Int J Syst Bacteriol. 1987;37:160–2. 10.1099/00207713-37-2-160.

[6] Zimmerman JJ, Karriker LA, Ramirez A, Schwartz KJ, Stevenson GW, Zhang J. In: Gottschalk M, Segura M, editors. Diseases of Swine—Streptococcosis. 11th ed. Hoboken: Wiley; 2019. p. 934–50.

[7] Quinn PJ, Markey BK, Leonard FC, FitzPatric ES, Fanning S, Hartigan PJ. Veterinary Microbiology and Microbial Disease Streptococci. 2nd ed. Hoboken: Wiley; 2011. p. 188–95.

[8] MacInnes JI, Gottschalk M, Lone AG, Metcalf DS, Ojha S, Rosendal T, et al. Prevalence of Actinobacillus pleuropneumoniae, Actinobacillus suis, Haemophilus parasuis, Pasteurella multocida, and Streptococcus suis in representative Ontario swine herds. Can J Vet Res. 2008;72:242–8. 18505187 PMC2327245

[9] Amass SF, Clark LK, Wu CC. Source and timing of Streptococcus suis infection in neonatal pigs: implications for early weaning procedures. J Swine Health Prod. 1995;3:189–93.

[10] Berthelot-Hérault F, Gottschalk M, Labbé A, Cariolet R, Kobisch M. Experimental airborne transmission of Streptococcus suis capsular type 2 in pigs. Vet Microbiol. 2001;82:69–80. 11423197 10.1016/s0378-1135(01)00376-5

[11] Dee SA, Carlson AR, Winkelman NL, Corey MM. Effect of management practices on the Streptococcus suis carrier rate in nursery swine. J Am Vet Med Assoc. 1993;203:295–9. 8407494

[12] Torremorell M, Pijoan C. Prolonged persistence of an epidemic Streptococcus suis strain in a closed pig population. Vet Rec. 1998;143:394–5. 9802198 10.1136/vr.143.14.394

[13] Higgins R, Gottschalk M. Distribution of Streptococcus suis capsular types in 1999. Can Vet J. 2000;41:414. 10816840 PMC1476256

[14] Okura M, Osaki M, Nomoto R, Arai S, Osawa R, Sekizaki T, et al. Current taxonomical situation of Streptococcus suis. Pathogens. 2016;5:45. 27348006 10.3390/pathogens5030045 PMC5039425

[15] Chatellier S, Harel J, Zhang Y, Gottschalk M, Higgins R, Devriese LA, et al. Phylogenetic diversity of Streptococcus suis strains of various serotypes as revealed by 16S rRNA gene sequence comparison. Int J Syst Evol Microbiol. 1998;48:581–9. 10.1099/00207713-48-2-581. 10.1099/00207713-48-2-581 9731300

[16] Hill JE, Gottschalk M, Brousseau R, Harel J, Hemmingsen SM, Goh SH. Biochemical analysis, cpn60 and 16S rDNA sequence data indicate that Streptococcus suis serotypes 32 and 34, isolated from pigs, are Streptococcus orisratti. Vet Microbiol. 2005;107:63–9. 15795078 10.1016/j.vetmic.2005.01.003

[17] Tien LHT, Nishibori T, Nishitani Y, Nomoto R, Osawa R. Reappraisal of the taxonomy of Streptococcus suis serotypes 20, 22, 26, and 33 based on DNA-DNA homology and sodA and recN phylogenies. Vet Microbiol. 2013;162:842–9. 23245487 10.1016/j.vetmic.2012.11.001

[18] Nomoto R, Maruyama F, Ishida S, Tohya M, Sekizaki T, Osawa R. Reappraisal of the taxonomy of Streptococcus suis serotypes 20, 22 and 26: Streptococcus parasuis sp. nov. Int J Syst Evolut Microbiol. 2015;65:438–43. 10.1099/ijs.0.067116-0. 10.1099/ijs.0.067116-0 25385995

[19] Pan Z, Ma J, Dong W, Song W, Wang K, Lu C, et al. Novel variant serotype of Streptococcus suis isolated from piglets with meningitis. Appl Environ Microbiol. 2015;81:976–85. 25416757 10.1128/AEM.02962-14 PMC4292476

[20] Okura M, Auger JP, Shibahara T, Goyette-Desjardins G, Van Calsteren MR, Maruyama F, et al. Capsular polysaccharide switching in Streptococcus suis modulates host cell interactions and virulence. Sci Rep. 2021;11:6513. 33753801 10.1038/s41598-021-85882-3 PMC7985379

[21] Goyette-Desjardins G, Auger JP, Xu J, Segura M, Gottschalk M. Streptococcus suis, an important pig pathogen and emerging zoonotic agent-an update on the worldwide distribution based on serotyping and sequence typing. Emerg Microbes Infect. 2014;3: e45. 26038745 10.1038/emi.2014.45 PMC4078792

[22] Li K, Lacouture S, Lewandowski E, Thibault E, Gantelet H, Gottschalk M, et al. Molecular characterization of Streptococcus suis isolates recovered from diseased pigs in Europe. Vet Res. 2024;55:117. 10.1186/s13567-024-01366-y. 39334446 10.1186/s13567-024-01366-y PMC11429987

[23] Kataoka Y, Sugimoto C, Nakazawa M, Morozumi T, Kashiwazaki M. The epidemiological studies of Streptococcus suis infections in Japan from 1987 to 1991. J Vet Med Sci. 1993;55:623–6. 8399744 10.1292/jvms.55.623

[24] Aarestrup FM, Jorsal SE, Jensen NE. Serological characterization and antimicrobial susceptibility of Streptococcus suis isolates from diagnostic samples in Denmark during 1995 and 1996. Vet Microbiol. 1998;60:59–66. 9595627 10.1016/s0378-1135(98)00147-3

[25] Wisselink HJ, Smith HE, Stockhofe-Zurwieden N, Peperkamp K, Vecht U. Distribution of capsular types and production of muramidase-released protein (MRP) and extracellular factor (EF) of Streptococcus suis strains isolated from diseased pigs in seven European countries. Vet Microbiol. 2000;74:237–48. 10808092 10.1016/s0378-1135(00)00188-7

[26] Petrocchi-Rilo M, Martínez-Martínez S, Aguarón-Turrientes Á, Roca-Martínez E, García-Iglesias MJ, Pérez-Fernández E, et al. Anatomical site, typing, virulence gene profiling, antimicrobial susceptibility and resistance genes of Streptococcus suis isolates recovered from pigs in Spain. Antibiotics (Basel). 2021;10:707. 34208248 10.3390/antibiotics10060707 PMC8230935

[27] Lacouture S, Olivera YR, Mariela S, Gottschalk M. Distribution and characterization of Streptococcus suis serotypes isolated from January 2015 to June 2020 from diseased pigs in Québec. Canada Can J Vet Res. 2022;86:78–82. 34975227 PMC8697323

[28] Gottschalk M, Lacouture S. Canada: Distribution of Streptococcus suis (from 2012 to 2014) and Actinobacillus pleuropneumoniae (from 2011 to 2014) serotypes isolated from diseased pigs. Can Vet J. 2015;56:1093–4. 26483588 PMC4572831

[29] Gottschalk M, Lacouture S, Bonifait L, Roy D, Fittipaldi N, Grenier D. Characterization of Streptococcus suis isolates recovered between 2008 and 2011 from diseased pigs in Québec, Canada. Vet Microbiol. 2013;162:819–25. 23177911 10.1016/j.vetmic.2012.10.028

[30] Zouharová M, Šimek B, Gebauer J, Králová N, Kucharovičová I, Plodková H, et al. Characterisation of Streptococcus suis isolates in the Czech republic collected from diseased pigs in the years 2018–2022. Pathogens. 2022;12:5. 36678353 10.3390/pathogens12010005 PMC9862946

[31] Nghia HDT, Hoa NT, Linh LD, Campbell J, Diep TS, Chau NVV, et al. Human case of Streptococcus suis serotype 16 infection. Emerg Infect Dis. 2008;14:155–7. 18258097 10.3201/eid1401.070534 PMC2600150

[32] Gottschalk M, Higgins R, Boudreau M. Use of polyvalent coagglutination reagents for serotyping of Streptococcus suis. J Clin Microbiol. 1993;31:2192–4. 8370749 10.1128/jcm.31.8.2192-2194.1993 PMC265720

[33] Cloutier G, D’Allaire S, Martinez G, Surprenant C, Lacouture S, Gottschalk M. Epidemiology of *Streptococcus suis* serotype 5 infection in a pig herd with and without clinical disease. Vet Microbiol. 2003;97:135–51. 14637045 10.1016/j.vetmic.2003.09.018

[34] Kerdsin A, Akeda Y, Hatrongjit R, Detchawna U, Sekizaki T, Hamada S, et al. Streptococcus suis serotyping by a new multiplex PCR. J Med Microbiol. 2014;63:824–30. 24696517 10.1099/jmm.0.069757-0

[35] Baums CG, Verkühlen GJ, Rehm T, Silva LMG, Beyerbach M, Pohlmeyer K, et al. Prevalence of Streptococcus suis genotypes in wild boars of Northwestern Germany. Appl Environ Microbiol. 2007;73:711–7. 17085699 10.1128/AEM.01800-06 PMC1800741

[36] Palmieri C, Varaldo PE, Facinelli B. Streptococcus suis, an emerging drug-resistant animal and human pathogen. Front Microbiol. 2011. 10.3389/fmicb.2011.00235/full. 22275909 10.3389/fmicb.2011.00235 PMC3223616

[37] Zheng H, Du P, Qiu X, Kerdsin A, Roy D, Bai X, et al. Genomic comparisons of Streptococcus suis serotype 9 strains recovered from diseased pigs in Spain and Canada. Vet Res. 2018;49:1. 29316972 10.1186/s13567-017-0498-2 PMC5759227

[38] Princivalli MS, Palmieri C, Magi G, Vignaroli C, Manzin A, Camporese A, et al. Genetic diversity of Streptococcus suis clinical isolates from pigs and humans in Italy (2003–2007). Eurosurveillance. 2009. 10.2807/ese.14.33.19310-en. 19712640 10.2807/ese.14.33.19310-en

[39] Varela NP, Gadbois P, Thibault C, Gottschalk M, Dick P, Wilson J. Antimicrobial resistance and prudent drug use for Streptococcus suis. Animal Health Res Rev. 2013;14:68–77. 10.1017/S1466252313000029 23683342

[40] Vela AI, MiguelA M, Cebolla JA, González S, Latre MV, Domínguez L, et al. Antimicrobial susceptibility of clinical strains of *Streptococcus suis* isolated from pigs in Spain. Vet Microbiol. 2005;105:143–7. 15627526 10.1016/j.vetmic.2004.10.009

[41] Holden MTG, Hauser H, Sanders M, Ngo TH, Cherevach I, Cronin A, et al. Rapid evolution of virulence and drug resistance in the emerging zoonotic pathogen Streptococcus suis. PLoS ONE. 2009;4: e6072. 19603075 10.1371/journal.pone.0006072 PMC2705793

[42] Hu P, Yang M, Zhang A, Wu J, Chen B, Hua Y, et al. Comparative genomics study of multi-drug-resistance mechanisms in the antibiotic-resistant Streptococcus suis R61 strain. PLoS ONE. 2011;6:e24988. 10.1371/journal.pone.0024988. 21966396 10.1371/journal.pone.0024988 PMC3180280

[43] Zhang C, Ning Y, Zhang Z, Song L, Qiu H, Gao H. In vitro antimicrobial susceptibility of *Streptococcus suis* strains isolated from clinically healthy sows in China. Vet Microbiol. 2008;131:386–92. 18499362 10.1016/j.vetmic.2008.04.005

[44] Wisselink HJ, Veldman KT, Van den Eede C, Salmon SA, Mevius DJ. Quantitative susceptibility of *Streptococcus suis* strains isolated from diseased pigs in seven European countries to antimicrobial agents licenced in veterinary medicine. Vet Microbiol. 2006;113:73–82. 16387456 10.1016/j.vetmic.2005.10.035

[45] Aarestrup FM, Rasmussen SR, Artursson K, Jensen NE. Trends in the resistance to antimicrobial agents of *Streptococcus suis* isolates from Denmark and Sweden1. Vet Microbiol. 1998;63:71–80. 9810623 10.1016/s0378-1135(98)00228-4

[46] Takamatsu D, Osaki M, Sekizaki T. Chloramphenicol resistance transposable element Tn*Ss1* of *Streptococcus suis*, a transposon flanked by IS*6*-family elements. Plasmid. 2003;49:143–51. 12726767 10.1016/s0147-619x(02)00149-x

[47] Du F, Lv X, Duan D, Wang L, Huang J. Characterization of a linezolid- and vancomycin-resistant Streptococcus suis isolate that harbors optrA and vanG operons. Front Microbiol. 2019. 10.3389/fmicb.2019.02026/full. 31551963 10.3389/fmicb.2019.02026 PMC6746840

[48] Segura M, Aragon V, Brockmeier SL, Gebhart C, Greeff A de, Kerdsin A, et al. Update on Streptococcus suis research and prevention in the era of antimicrobial restriction. In: 4th International Workshop on S. suis. Pathogens. 2020;9:374 10.3390/pathogens9050374 PMC7281350 32422856

[49] Huang J, Ma J, Shang K, Hu X, Liang Y, Li D, et al. Evolution and diversity of the antimicrobial resistance associated mobilome in Streptococcus suis: a probable mobile genetic elements reservoir for other Streptococci. Front Cell Infect Microbiol. 2016. 10.3389/fcimb.2016.00118. 27774436 10.3389/fcimb.2016.00118 PMC5053989

[50] Hernandez-Garcia J, Wang J, Restif O, Holmes MA, Mather AE, Weinert LA, et al. Patterns of antimicrobial resistance in Streptococcus suis isolates from pigs with or without streptococcal disease in England between 2009 and 2014. Vet Microbiol. 2017;207:117–24. 28757010 10.1016/j.vetmic.2017.06.002 PMC5548070

[51] Lacouture S, Okura M, Takamatsu D, Corsaut L, Gottschalk M. Development of a mismatch amplification mutation assay to correctly serotype isolates of Streptococcus suis serotypes 1, 2, 1/2, and 14. J Vet Diagn Invest. 2020;32:490–4. 32306861 10.1177/1040638720915869 PMC7377628

[52] Ishida S, Tien LHT, Osawa R, Tohya M, Nomoto R, Kawamura Y, et al. Development of an appropriate PCR system for the reclassification of Streptococcus suis. J Microbiol Methods. 2014;107:66–70. 25229648 10.1016/j.mimet.2014.09.003

[53] Obradovic MR, Segura M, Segalés J, Gottschalk M. Review of the speculative role of co-infections in Streptococcus suis-associated diseases in pigs. Vet Res. 2021;52:49. 33743838 10.1186/s13567-021-00918-w PMC7980725

[54] Liu P, Zhang Y, Tang H, Wang Y, Sun X. Prevalence of Streptococcus suis in pigs in China during 2000–2021: a systematic review and meta-analysis. One Health. 2023;16:100513. 37363255 10.1016/j.onehlt.2023.100513 PMC10288055

[55] Flores JLM, Higgins R, D’Allaire S, Charette R, Boudreau M, Gottschalk M. Distribution of the different capsular types of Streptococcus suis in nineteen swine nurseries. Can Vet J. 1993;34:170–1. 17424186 PMC1686519

[56] Cucco L, Paniccià M, Massacci FR, Morelli A, Ancora M, Mangone I, et al. New sequence types and antimicrobial drug-resistant strains of Streptococcus suis in diseased pigs, Italy, 2017–2019. Emerg Infect Dis. 2022;28:139–47. 34932464 10.3201/eid2801.210816 PMC8714200

[57] Scherrer S, Rosato G, Spoerry Serrano N, Stevens MJA, Rademacher F, Schrenzel J, et al. Population structure, genetic diversity and pathotypes of Streptococcus suis isolated during the last 13 years from diseased pigs in Switzerland. Vet Res. 2020;51:85. 32641158 10.1186/s13567-020-00813-w PMC7346511

[58] Amass SF, Clark LK, Knox K, Wu CC, Hill MA. Streptococcus suis colonization of piglets during parturition. J Swine Health Product. 1996;4:269–72.

[59] Blume V, Luque I, Vela AI, Borge C, Maldonado A, Domínguez L, et al. Genetic and virulence-phenotype characterization of serotypes 2 and 9 of Streptococcus suis swine isolates. Int Microbiol. 2009;12:161–6. 19784922

[60] Dolbec D, Lehoux M, Okura M, Takamatsu D, Gottschalk M, Segura M. Streptococcus suis surface-antigen recognition by antibodies and bacterial elimination is influenced by capsular polysaccharide structure. Front Cell Infect Microbiol. 2023. 10.3389/fcimb.2023.1228496/full. 37545852 10.3389/fcimb.2023.1228496 PMC10401424

[61] MacInnes JI, Desrosiers R. Agents of the “suis-ide diseases” of swine: Actinobacillus suis, Haemophilus parasuis, and Streptococcus suis. Can J Vet Res. 1999;63:83–9. 10369563 PMC1189524

[62] Lapointe L, D’Allaire S, Lebrun A, Lacouture S, Gottschalk M. Antibody response to an autogenous vaccine and serologic profile for Streptococcus suis capsular type 1/2. Can J Vet Res. 2002;66:8–14. 11858652 PMC226975

[63] Meekhanon N, Kaewmongkol S, Phimpraphai W, Okura M, Osaki M, Sekizaki T, et al. Potentially hazardous Streptococcus suis strains latent in asymptomatic pigs in a major swine production area of Thailand. J Med Microbiol. 2017;66:662–9. 28516843 10.1099/jmm.0.000483

[64] Lunha K, Chumpol W, Samngamnim S, Jiemsup S, Assavacheep P, Yongkiettrakul S. Antimicrobial susceptibility of Streptococcus suis isolated from diseased pigs in Thailand, 2018–2020. Antibiotics. 2022;11 10.3390/antibiotics11030410 PMC8944821 35326873

[65] Tan M-F, Tan J, Zeng Y-B, Li H-Q, Yang Q, Zhou R. Antimicrobial resistance phenotypes and genotypes of Streptococcus suis isolated from clinically healthy pigs from 2017 to 2019 in Jiangxi Province China. J Appl Microbiol. 2021;130:797–806. 32881196 10.1111/jam.14831

[66] Yongkiettrakul S, Maneerat K, Arechanajan B, Malila Y, Srimanote P, Gottschalk M, et al. Antimicrobial susceptibility of Streptococcus suis isolated from diseased pigs, asymptomatic pigs, and human patients in Thailand. BMC Vet Res. 2019;15:5. 30606175 10.1186/s12917-018-1732-5 PMC6318959

